# Effects of high intensity interval training on exercise capacity in people with cystic fibrosis: study protocol for a randomised controlled trial

**DOI:** 10.1186/s13102-018-0108-2

**Published:** 2018-11-06

**Authors:** Abbey Sawyer, Vinicius Cavalheri, Sue Jenkins, Jamie Wood, Nola Cecins, Bhajan Singh, Kylie Hill

**Affiliations:** 1 School of Physiotherapy and Exercise Science, Faculty of Health Science, Curtin University, GPO Box U1987, Perth, WA 6845 Australia; 2 Physiotherapy Department, Sir Charles Gairdner Hospital, Perth, WA Australia; 3 Institute for Respiratory Health, Perth, WA Australia; 4 Department of Pulmonary Physiology and Sleep Medicine, Sir Charles Gairdner Hospital, Perth, WA Australia; 5 West Australian Sleep Disorders Research Institute, Nedlands, WA Australia; 6 Faculty of Science, University of Western Australia, Crawley, WA Australia

**Keywords:** Cystic fibrosis, Exercise, High intensity interval training

## Abstract

**Background:**

In people with cystic fibrosis (CF), higher exercise capacity is associated with better health-related quality of life (HRQoL), reduced risk of hospitalisation for a respiratory infection and survival. Therefore, optimisation of exercise capacity is an important treatment goal. The Australian and New Zealand clinical practice guidelines recommend that people with CF complete 30 to 60 min of moderate intensity aerobic exercise on most days of the week. This recommendation can be difficult to achieve by people with CF because of time constraints, and intolerable breathlessness and muscle fatigue during continuous exercise. In contrast, a low-volume, high intensity interval training (HIIT) program may be a more achievable and efficient training method to improve exercise capacity in people with CF.

**Methods:**

A randomised controlled trial will be undertaken. Forty people with CF (aged ≥15 years) will be randomly allocated, on a 1:1 ratio, to either the experimental or control group. Regardless of their group allocation, all participants will be asked to continue with their usual daily treatment for the study duration. Those in the experimental group will complete 8 weeks of thrice weekly HIIT on a cycle ergometer. Those in the control group will receive weekly contact with the investigators. The primary outcome of this study is exercise capacity. Secondary outcomes are HRQoL, exercise self-efficacy, feelings of anxiety, depression and enjoyment. These outcomes will be recorded at baseline (i.e. prior to randomisation) and following the 8-week intervention period. The study will also report other outcomes of the HIIT program (cardiovascular responses, symptom response, post-exercise muscle soreness and tolerance) and behaviour change techniques such as reinforcement, feedback and goal setting, used during the HIIT program.

**Discussion:**

This study will determine the effects of 8-weeks of supervised, low-volume HIIT, completed on a cycle ergometer on measures of exercise capacity, HRQoL, exercise self-efficacy, feelings of anxiety, depression and enjoyment. If effective, this type of training could be an attractive alternative to traditional continuous training because it may be more achievable and time efficient.

**Trial registration:**

Australian and New Zealand Clinical Trials Registry (ANZCTR):12617001271392 (04/09/2017).

## Background

Cystic fibrosis (CF) is a genetic disease which predominantly affects Caucasians [1]. Several organs are affected by CF and premature death, primarily due to respiratory failure, is inevitable [1]. Advancements in multidisciplinary care over the past 50 years have resulted in a rise in median life expectancy from 1 year in 1938 [2, 3], 10 years in 1966, 28 years in 1989 [4] to now over 40 years of age [5]. These advancements in multidisciplinary care come at the cost of a high daily treatment burden. That is, the medical, nutritional and physiotherapy regimens for people with CF are time consuming and can take up to 4 h each day [6], which need to be accommodated with the usual demands of life, such as work, study and family commitments [7].

Compared to their healthy counterparts, important markers of exercise capacity are reduced in people with CF [8, 9]. The cause of such reduction is multifactorial and includes decrements in lung function, peripheral muscle deconditioning which are both likely to contribute to severe breathlessness during exercise [8]. Additionally, people with CF may have impaired cardiac and vascular function [10–12]. In this population, higher exercise capacity, for example the peak rate of oxygen uptake (VO_2peak_), is associated with better health-related quality of life (HRQoL) [13, 14], reduced risk of hospitalisation due a respiratory infection [15] and survival [16, 17]. Given this relationship, improving exercise capacity is an important treatment goal in this population [18].

Markers of exercise capacity can be improved in people with CF by undertaking ‘traditional’ moderate intensity, continuous, aerobic exercise training [19–22]. While the optimal method of exercise training in this population requires further investigation [23], the Australian and New Zealand clinical practice guidelines recommend that people with CF complete 30 to 60 min of moderate intensity exercise on most days of the week [24]. However, in people with CF, this type of exercise training is burdensome, adding to the already high daily treatment burden, and may not be the most appropriate option due to several factors, including a ‘lack of time’ [25] and intolerable symptoms of breathlessness and muscle fatigue experienced during continuous exercise [26]. Furthermore, oxygen desaturation is more likely during moderate intensity continuous exercise [27], and this type of training may augment inflammation in this population compared to interval training [28]. An alternative training approach, such as low-volume high intensity interval training (HIIT), may be a more achievable and efficient method to optimise exercise capacity in this population [29].

High intensity interval training consists of short periods of high intensity exercise (‘work’), interrupted by periods of lower intensity exercise (‘rest’) [30]. The ‘rest’ period allows for partial recovery of symptoms such as breathlessness and muscle fatigue, and therefore offers the opportunity to optimise the training intensity achieved during the ‘work’ periods [31, 32]. In healthy adults [32] and people with chronic obstructive pulmonary disease (COPD) [33], HIIT is well tolerated and offers similar gains in exercise capacity to continuous training, with fewer symptoms of breathlessness and muscle fatigue [34, 35], and lower training time [36] or work [37] per session. Additionally, HIIT has been reported to be more enjoyable than other modes of exercise training [38]. Preliminary data suggest that HIIT is feasible in people with CF [26, 27]. However, the effectiveness of low-volume HIIT to improve exercise capacity in this population has not been investigated in a randomised controlled trial (RCT).

The primary aim of this RCT is, in people with CF aged ≥15 years, to determine the effects of an 8-week supervised low-volume HIIT program on exercise capacity (primary outcome), and HRQoL, exercise self-efficacy, feelings of anxiety, depression and enjoyment. As a secondary aim, in those allocated to receive HIIT, this study will also report on: (i) the proportion of participants who develop post-exercise quadriceps femoris muscle soreness each week during the 8-week HIIT program as well as the severity of this symptom; (ii) the participant tolerance of the 8-week supervised HIIT program; (iii) the cardiorespiratory and symptom responses elicited during the HIIT sessions throughout the 8-week program and; (iv) the behaviour change techniques (BCTs) employed throughout the program. If the HIIT program is shown to be effective at improving exercise capacity, the implementation of such a time efficient program will result in a lower treatment burden, compared to undertaking moderate intensity continuous exercise and therefore may be more readily incorporated into the daily routine of people with CF.

## Methods

**Fig. 1:**
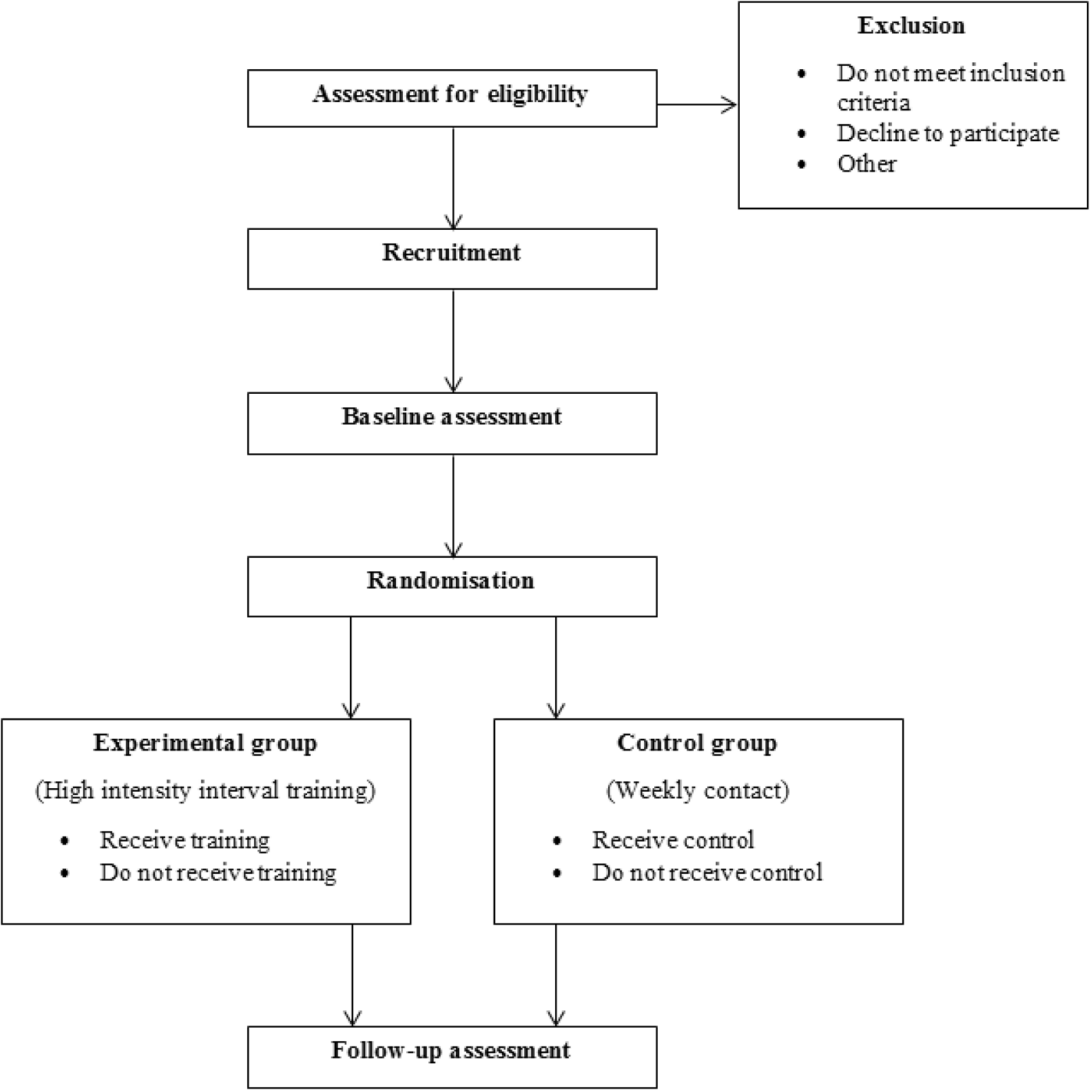
Study design flow diagram

This is a prospective, single-blinded RCT. The study has been approved by the relevant ethics committees and has been registered with the Australian and New Zealand Clinical Trials Registry (ANZCTR) (12617001271392, 04/09/2017). Participant recruitment will commence in September 2017. The study will be reported in accordance with the Consolidated Standards of Reporting Trials (CONSORT) statement [39]. The study flow diagram is presented in Fig. 1.

### Recruitment and consent

Potential participants will be identified and recruited from scheduled outpatient clinic appointments at the adult (Sir Charles Gairdner Hospital [SCGH]) and children’s (Perth Children’s Hospital ([PCH]) CF centres. Potential participants will be provided with an information sheet summarising the study and will receive a phone call within 48 h of their clinic visit to discuss their willingness to participate. At the beginning of the first study visit, written informed consent will be obtained from participants or assent if the participant is under 18 years of age.

#### Inclusion and exclusion criteria

Males and females with CF will be invited to participate in this study if they: (i) are aged ≥15 years and; (ii) have a body mass index (BMI) > 16 kg/m^2^. Exclusion criteria will comprise: (i) current or recent (within the previous 4 weeks) exacerbation of CF which required oral or intravenous antibiotics; (ii) a co-morbid condition that would impact on the ability to undertake a maximal incremental exercise capacity test; (iii) poorly controlled diabetes as deemed by their treating endocrinologist; (iv) previous lung transplant or current listing for lung transplantation; (v) participation in exercise at a moderate intensity two or more times per week for the previous 3 months and; (vi) the inability to provide written informed consent due to a cognitive impairment or being unable to understand English. If a potential participant has cystic fibrosis-related diabetes, they will be included pending approval from the treating endocrinologist, irrespective of the treatment they are on.

### Randomisation and allocation concealment

Participants will be randomly allocated, on a 1:1 ratio, to either the experimental or control group. Concealment of the allocation sequence will be ensured by using a central randomisation service (the National Health and Medical Research Council randomisation service). A minimisation algorithm will be used to stratify for site of recruitment (i.e. SCGH or PCH), spirometry (i.e. mild [FEV_1_ ≥ 70% predicted], moderate [FEV_1_ 40 to 69% predicted] or severe [FEV_1_ ≤ 39% predicted]) and the use (or not) of Ivacaftor, which is a medication that improves lung function, weight, HRQoL and reduces the rate of exacerbation [40].

### Assessment period

Both prior to randomisation (i.e.baseline) and following the intervention period (i.e. follow-up), participants will complete assessments over two non-consecutive days; assessment day one and assessment day two. Each assessment visit will last 1.5 h, and both visits will be completed within 2 weeks. During both the baseline and follow-up assessments, descriptive variables will be recorded related to age, gender, height, weight, genotype and spirometry (Medgraphics USB Spirometer, MCG Diagnostics, Minnesota, USA). Measures related to the primary aim will be collected in both groups during the baseline and follow-up assessment. This will comprise exercise capacity, HRQoL, exercise self-efficacy, feelings of anxiety, depression and enjoyment. The follow-up assessments of these outcomes will be completed by an assessor who is blinded to group allocation. Measures related to the secondary aim will be collected in the experimental group only and will comprise post-exercise muscle soreness (measured weekly), tolerance (measured at every HIIT session) and cardiorespiratory and symptom responses to HIIT (measured at week 1, week 4 and week 8 of the HIIT program) will be measured throughout the 8-week period. Details of these measurements are provided below.

#### Assessment day one

On the first assessment day each participant will complete a ramp-based cycle ergometry test on an electronically-braked cycle ergometer (Ergoselect 100; Ergoline, Bitz, Germany). The test will commence with 1 min of rest, followed by 1 min of unloaded cycling. Thereafter, a ‘continuous ramp’ protocol will be used to progressively increase the work rate until the participant is unable to continue due to intolerable symptoms (i.e. symptom limitation). The magnitude of change in work rate will be individualised based on the participant’s age and pre-existing level of fitness (as subjectively reported by the participant), with the aim of achieving symptom limitation in 8 to 12 min [41]. Throughout the test, participants will be asked to cycle at a cadence of 60 rpm. Breath by breath measurements will be collected of minute ventilation, breathing pattern, rate of oxygen consumption (VO_2_) and rate of expired carbon dioxide production (VCO_2_) (Medgraphics CardioO_2_; Medical Graphics Corporation). Measures of heart rate (HR) and oxygen saturation (SpO_2_) will be continuously monitored and recorded using a 12 lead electrocardiogram and an ear probe attached to a pulse oximeter (Ohmeda Biox 3700e ear probe, Colorado, USA), respectively. Blood pressure will be measured every 2 min using an automated blood pressure (BP) machine (Tango M2; Suntech, North Carolina, USA) with a BP cuff connected to the participants’ right arm. Measures of breathlessness and muscle fatigue will be recorded each minute using a modified Borg scale [42]. The peak rate of oxygen uptake (VO_2peak_) will be defined as the average VO_2_ during the final 30 s of the test [43].

Following completion of the ramp-based cycle ergometry test, the participants will be asked to complete two questionnaires: (i) the Cystic Fibrosis Questionnaire- Revised (CFQ-R) [44, 45] which takes approximately 15 min to complete and is a valid and reliable questionnaire for use in people with CF [44, 45], and; (ii) the Barriers Self-efficacy Scale (BARSE) [46], which takes approximately 5 min to complete and is a valid and reliable tool in healthy people [46].

#### Assessment day two

On a separate, non-consecutive day, the constant work rate test will be performed in order to establish time to symptom limitation (T_lim_) (primary outcome). This test will be conducted using identical equipment and measurements as described for the ramp-based cycle-ergometry test. Participants will be asked to complete a 1-min warm-up of unloaded cycling after which the work rate will be increased to 80% of the W_max_ [48]. An intensity of 80% of the W_max_ has been chosen as this has been demonstrated to be feasible in people with CF [48]. In addition, at this cycling intensity, more than half of people with chronic respiratory disease (CF or COPD) achieve a T_lim_ of between 8 and 11 min; a test duration which is most responsive to change following the intervention period [49, 50]. During the baseline assessment, if a participant does not demonstrate signs of symptom limitation at 10 min, then the test will be terminated and, following a seated rest of 30 min, the test will be repeated at a higher intensity [43, 50]. During the follow-up assessment, the constant work rate test will be completed at the highest work rate used during the baseline assessment. In the follow-up assessment, the constant work rate test will be terminated at 20 min for participants who do not reach symptom limitation earlier and T_lim_ will be recorded as 20 min.

Following completion of the constant work rate exercise test, participants will be asked to complete three questionnaires; (i) the Alfred Wellness Score for CF (AweScore-CF) [51], which takes approximately 2 min to complete and is a reliable tool for use in people with CF [51]; (ii) the Hospital Anxiety and Depression Scale (HADS) [52], which takes approximately 10 min to complete, is a valid, reliable and responsive measure and has been widely used in people with CF [52], and; (iii) the Physical Activity Enjoyment Scale (PACES), which is a valid and reliable tool previously used in healthy people and takes approximately 5 min to complete [53].

### Intervention period

Regardless of group allocation, throughout the duration of the study, participants will continue usual care. This includes medication, nutritional support, airway clearance regimens and attendance at a multidisciplinary outpatient CF clinic, which occur quarterly, or more frequently if clinically indicated. In both groups, usual care for each participant will be recorded weekly throughout the intervention period. To do so, participants will be asked if there have been any changes to their treatment in the preceding week.

#### Experimental group

Participants allocated to the experimental group will be asked to complete an 8-week cycling-based HIIT program. Each training session will involve a 2-min active ‘warm up’ at 15 to 20 W, followed by a 30-s ‘work’ phase and 30-s ‘rest’ period, repeated six times. The work to rest periods will be followed by a 2-min active ‘cool down’ period at 15 to 20 W. Therefore, the total training time per session, inclusive of rest periods, will be 10 min. Each session will be supervised by a physiotherapist who is trained in the management of people with CF. To minimise the onset of post-exercise muscle soreness and optimise adherence, the training program will commence with a ‘lead in’ phase which will involve only two sessions of HIIT in weeks 1 and 2. Thereafter, between weeks 3 and 8, HIIT will be undertaken three times a week (i.e. total 22 sessions over 8 weeks). The training intensity will be prescribed using measurements of maximum work rate (W_max_) achieved during the ramp-based cycle ergometry test completed during the baseline assessments. Specifically, the first training session will be prescribed at 60% of W_max_, with the goal of achieving a training intensity equal to 80% of W_max_ during the fourth training session (i.e. the end of week 2). Thereafter, training intensity will be increased as rapidly as symptoms of breathlessness and muscle fatigue permit. Training will be completed at one of two sites, with the participant permitted to choose the site that is most convenient for them. The model of exercise bike used for the HIIT sessions will be identical across all training sites (Orbit Eco Generator Interval Bike OEB2002, Perth, Australia). Only a single participant will be permitted to use the exercise room on any day, with stringent hand hygiene and cleaning procedures adhered to at all times to reduce the risk of cross contamination between participants [54]. The intervention will be conducted and reported in accordance with the TIDieR checklist [55] and CERT guidelines [56, 57].

If a participant in the experimental group reports the onset of symptoms indicative of an exacerbation of CF (i.e. increased sputum volume, changes in the characteristics of sputum, blood present in the sputum, increased cough, pain from coughing, new wheeze or increased wheeze, new or increased chest tightness, shortness of breath or difficulty breathing, increased fatigue or lethargy, fever, loss of appetite or weight, sinus pain or tenderness) [58], they will be referred to their respective CF team for medical review. If an exacerbation is diagnosed by the treating CF team, the participant will be invited to continue participating in HIIT sessions once deemed to be medically stable by a CF clinician. Medical stability has been defined as being afebrile, having resting BP, HR and SpO_2_ within limits of the participants’ baseline values and/or deemed stable by the treating CF clinician. In the event of missed attendance to HIIT sessions, the training program will be extended by a maximum of 2 weeks so that the participant will have 10 weeks to complete the 22 HIIT sessions.

#### Measurements related to the secondary aim (collected in the experimental group only)

##### Post-exercise muscle soreness

Participants in the experimental group will be asked if they experienced any post-exercise quadriceps femoris muscle soreness whilst completing a ‘sit to stand’ 24 h following the first training session of each week. Those who report experiencing post-exercise muscle soreness will be asked to rate its severity using a Visual Analogue Scale (VAS) [59].

##### Tolerance

The level of participant attendance and completion of the HIIT sessions will be recorded throughout the 8-week period. Oxygen saturation (SpO_2_) at rest and nadir SpO_2_ will be recorded during each training session. Adverse events will be monitored throughout the HIIT program. These will be categorised as minor, if they are transient and self-limiting events (i.e. breathlessness without significant oxygen desaturation [< 4% from the participant’s pre-exercise SpO_2_], muscle or general fatigue) or major events if they require the participant to cease training during any given session or necessitate medical assistance (i.e. breathlessness with significant oxygen desaturation [≥ 4% from the participants pre-exercise SpO_2_], pain, vasovagal events or haemoptysis).

##### Cardiorespiratory and symptom responses

During weeks 1, 4 and 8 of the training period, participants in the experimental group will be asked to complete one of two or three (week 3 onwards) HIIT sessions in the same laboratory used to conduct the ramp-based cycle ergometry test. During these sessions, measurements will be collected of minute ventilation, breathing pattern, VO_2_ and VCO_2_, HR, SpO_2_ and BP. Breathlessness and muscle fatigue will be assessed using the modified Borg scale [42].

##### Behaviour change techniques

The HIIT sessions will be audio recorded over the course of the 8-week program. The purpose of this will be to allow for qualitative analysis and reporting of BCTs [60], as recommended by the CONSORT guidelines [61]. Behaviour change techniques are defined as the active component of an intervention that targets a specific behaviour. These techniques can be used alone, or in combination with another technique. Of note, to be considered a BCT, the intervention needs to be observable, able to be replicated, and designed to alter existing or stimulate new behaviour [60]. Some examples of BCTs are reinforcement, self-monitoring, feedback, problem solving, graded tasks and reward. The BCTs will be reported in accordance with the Taxonomy (v1) by Michie et al. [60]. All HIIT sessions will be audio recorded, unless the participant ‘opts-out’. This audio recording process will also allow for fidelity checking of the sessions if required by the relevant ethics committee.

#### Control group

Participants allocated to the control group will be contacted once a week by a physiotherapist to discuss changes to their symptoms, healthcare utilisation and participation in exercise over the preceding week. Participants will be allowed to choose the way in which this contact is made; phone calls, texts or emails. If a participant allocated to the control group reports any symptoms that are suggestive of an exacerbation, they will be referred to the CF team for medical review.

### Statistical analyses

The results of this study will be analysed according to the intention-to-treat principle using Statistical Package for the Social Sciences (SPSS) (Version 22, SPSS, Chicago, IL, USA). The distribution of data will be checked using frequency histograms. A *p* value of < 0.05 will be considered statistically significant. Audio recording of the HIIT sessions will be analysed using NVivo software.

To address differences between the experimental and control group, between-group differences in all outcomes (e.g. T_lim_, VO_2peak_, HRQoL, exercise self-efficacy, feelings of anxiety, depression and enjoyment) will be assessed using linear mixed models. Baseline measures will be used as a covariate and group allocations will be used as a fixed factor. Total in work done (i.e. training dose) will be considered in the analyses by entering this parameter as a random factor in the mixed effects model. To address the proportion of participants who develop post-exercise muscle soreness, the severity of post-exercise muscle soreness in those who develop it over the 8 week intervention period and tolerance of the HIIT program, descriptive statistics, such as frequency, mean and standard deviation (SD) or median and interquartile range will be used. To explore differences in measures of cardiorespiratory and symptom responses throughout the HIIT program, an analysis of variance (parametric) or a Friedman’s test (non-parametric data) will be used.

### Sample size calculations

Constant power tests have been widely used to demonstrate increases in exercise capacity following a period of exercise training (i.e. pulmonary rehabilitation) [43, 47], are also more likely to reflect performance of activities of daily living [62], as they are performed at a moderate intensity, compared to tests of peak exercise capacity [22, 43] and when conducted at 80% of the W_max_ are demonstrated to be highly repeatable in people with CF [63]. For these reasons, the sample size calculation for this study has been based on the measurements of T_lim_ during a constant work rate test, conducted at 80% of W_max_ [43]. There is limited information pertaining to the minimal clinically important difference (MCID) for the T_lim_ in people with CF. However, in people with COPD, the MCID for T_lim_ during a constant work rate test has been proposed to be 100 s [43]. In order to detect a between-group difference of 100 s (MCID for T_lim_ in COPD), with a SD of 99 s (largest of the 2 SDs reported for T_lim_ in a CF population that will be similar to that recruited in the study described in this proposal [48]) (α = 0.05 and 1-β = 0.8) a sample size of 16 per group (*n* = 32 in total) is required. This sample size has been inflated by 20% to account for possible loss to follow-up. Therefore, the recruitment target for this study will be 40.

## Discussion

This study will be the first RCT to evaluate the effects of HIIT on exercise capacity, HRQoL, exercise self-efficacy, feelings of anxiety, depression and enjoyment in people with CF. If 30 min of HIIT (i.e. three 10 min sessions) per week is demonstrated to increase exercise capacity, HRQoL, exercise self-efficacy and enjoyment, it will represent a more achievable and efficient method of exercise and thereby will overcome the barrier of ‘lack of time’ [25]. This study will create an initial, yet strong evidence base for HIIT in people with CF and has the ability to influence clinical practice.

## Abbreviations

ANZCTR: Australian New Zealand clinical trials registry
AweScore-CF: Alfred wellness score for cystic fibrosis
BARES: Barriers self-efficacy scale
BMI: Body mass index
BP: Blood pressure
CF: Cystic fibrosis
CFQ-R: Cystic fibrosis questionnaire revised
CONSORT: Consolidated standards of reporting trials
COPD: Chronic obstructive pulmonary disease
FEV_1_: Forced expiratory volume in one second
HADS: Hospital anxiety and depression scale
HIIT: High intensity interval training
HR: Heart rate
HRQoL: Health-related quality of life
MCID: Minimal clinically important difference
PACES: Physical activity enjoyment scale
PCH: Perth children’s hospital
RCT: Randomised controlled trial
SCGH: Sir Charles Gairdner Hospital
SD: Standard deviation
SpO_2_: Oxygen saturation measured using a pulse oximeter
SPSS: Statistical package for the social sciences
T_lim_: Time to symptom limitation
VAS: Visual analogue scale
VCO_2_: Rate of expired carbon dioxide production
VO_2_: Rate of oxygen consumption
VO_2peak_: Peak rate of oxygen uptake
W_max_: Maximal work rate
